# Why Fibromyalgia Criteria Keep Changing: A Systematic Review of Misclassification, Measurement, and Diagnostic Spillover

**DOI:** 10.5152/ArchRheumatol.2026.26498

**Published:** 2026-07-06

**Authors:** Ali Gür

**Affiliations:** Division of Algology, Department of Physical Medicine and Rehabilitation, Gaziantep University Faculty of Medicine, Gaziantep, Türkiye

**Keywords:** Chronic widespread pain, classification criteria, diagnostic criteria, fibromyalgia, misclassification, rheumatology

## Abstract

**Background/Aims:**

Fibromyalgia is diagnosed without a definitive biomarker, so classification and diagnostic criteria carry unusual clinical and epidemiologic weight. This Preferred Reporting Items for Systematic Reviews and Meta-Analyses (PRISMA) 2020–guided systematic review examined why fibromyalgia criteria keep changing and how those changes influence overdiagnosis, misdiagnosis, missed diagnosis, chronic widespread pain boundaries, comorbid rheumatic disease, sex ratios, vitamin D deficiency, and prevalence estimates.

**Materials and Methods:**

MEDLINE/PubMed, Scopus, and Web of Science Core Collection were searched from January 1, 1990 to March 4, 2026. Backward and forward citation tracking was added. Eligible records included criteria papers, validation and concordance studies, prevalence studies, health-system analyses, comorbidity studies, and digital or body-map measurement studies. Fifty-eight records were included in the qualitative synthesis. Quality Assessment of Diagnostic Accuracy Studies-2 (QUADAS-2) and Risk of Bias In Non-Randomized Studies of Interventions (ROBINS-I)-informed domains guided appraisal where appropriate.

**Results:**

Across criteria eras, revisions redistributed rather than eliminated diagnostic error. The 1990 criteria standardized research cohorts but depended on tender-point examination. The 2010/2011 criteria improved feasibility and symptom capture, yet increased vulnerability to regional-pain and high-distress misclassification. The 2016 revision introduced a generalized pain requirement and clarified that fibromyalgia can coexist with other disorders, improving boundary control but not resolving clinician-criteria discordance, sex-sensitive under-recognition, or comorbidity-related diagnostic delay. Prevalence estimates vary partly because criteria architecture, sampling frame, pain-map methods, and case-finding purpose differ across studies.

**Conclusion:**

The 2016 criteria remain useful, but they are not a complete clinical diagnostic system. Future work should not merely add another threshold; it should combine generalized pain verification, dimensional severity reporting, comorbidity-aware interpretation, sex-sensitive screening, and explicit separation of chronic widespread pain from fibromyalgia.

**Main Points**

- Fibromyalgia criteria have changed because each generation solved 1 measurement problem while exposing another.
- The 2016 criteria are clinically useful but should be treated as a structured aid, not as a stand-alone diagnostic machine.
- Overdiagnosis, misdiagnosis, and missed diagnosis arise from different mechanisms and require different safeguards.
- Chronic widespread pain is not equivalent to fibromyalgia; symptom severity, distribution, duration, and comorbidity context must be integrated.
- In comorbid inflammatory disease or vitamin D deficiency, fibromyalgia should be diagnosed positively when the phenotype persists after proportionate evaluation of treatable contributors.

## Introduction

Fibromyalgia occupies a difficult diagnostic space because it is common, disabling, multisymptomatic, and still not anchored by a single confirmatory biomarker. In routine rheumatology practice, the diagnosis is therefore made through pattern recognition supported by criteria, not by a decisive laboratory or imaging test. The American College of Rheumatology (ACR) 1990 criteria standardized research cohorts through chronic widespread pain and tender-point examination, but subsequent ACR 2010, 2011, and 2016 revisions moved the field toward symptom-based and generalized pain-based definitions.^1^^-^^4^

This movement was not merely terminological. It reflected a deeper shift in the understanding of fibromyalgia from a peripheral tenderness syndrome toward a broader nociplastic and centralized pain phenotype that includes fatigue, unrefreshing sleep, cognitive symptoms, autonomic complaints, and functional impairment.^5^^-^^8^ International Classification of Diseases, 11th Revisions (ICD-11) and International Association for the Study of Pain (IASP) chronic pain classifications have further emphasized that chronic widespread pain and nociplastic pain require clinical recognition even when tissue damage or classic neuropathic mechanisms are absent.^9^^-^^12^

The clinical problem is that classification criteria and diagnostic criteria are often used as if they were interchangeable. Classification criteria are designed to create comparable research cohorts; clinical diagnosis must also handle uncertainty, comorbidity, incomplete histories, sex-related presentation differences, and treatable mimics. This distinction is especially important in fibromyalgia, where the reference standard is not independent of clinician judgment.^13^

The current debate is no longer limited to whether fibromyalgia is “real” or whether tender points should be counted. The more relevant question is whether existing criteria reduce the 3 diagnostic harms that matter most: overdiagnosis, misdiagnosis, and missed diagnosis. Overdiagnosis occurs when regional pain, nonspecific distress, or untreated mimics are labeled as fibromyalgia. Misdiagnosis occurs when fibromyalgia is used as a shortcut that obscures inflammatory, endocrine, neurologic, or sleep-related disease. Missed diagnosis occurs when genuine fibromyalgia is overlooked, particularly in men, in patients with inflammatory rheumatic disease, or in patients whose symptoms are attributed entirely to vitamin D deficiency, obesity, mood disorder, or another comorbidity.

This review therefore asks a deliberately practical question: after the 2016 criteria, what still needs to be improved? A total of 58 eligible reports were synthesized, and the evidence regarding the adequacy of the 2016 criteria, the need for future criteria development, the distinction between fibromyalgia and chronic widespread pain, diagnostic delay in comorbid illness, the interpretation of vitamin D deficiency, sex ratio shifts, and the broad prevalence range reported across epidemiologic studies was examined.

## Methods

This systematic review was designed and reported according to PRISMA 2020.^14^ MEDLINE/PubMed, Scopus, and Web of Science Core Collection were searched from January 1, 1990 to March 4, 2026. Search terms combined fibromyalgia with criteria, classification, diagnosis, chronic widespread pain, generalized pain, symptom severity, Widespread Pain Index (WPI), Symptom Severity Scale (SSS), polysymptomatic distress, validation, agreement, misclassification, prevalence, sex, body map, comorbidity, vitamin D, and digital or machine-learning measurement terms. Backward and forward citation tracking was performed for criteria papers and major validation studies. The detailed search strategy, study-selection accounting, PRISMA accounting, risk-of-bias approach, and evidence map are provided in Supplementary File 1.

The review question was framed with a modified Population, Intervention/Exposure, Comparator, Outcomes and Study Design (PICOS) structure. The population included adults with fibromyalgia, suspected fibromyalgia, chronic widespread pain, or comorbid rheumatic disease in which fibromyalgia was assessed. The exposure or index construct was the criteria set, diagnostic pathway, or measurement strategy used. Comparators included alternative criteria versions, clinician diagnosis, chronic widespread pain definitions, or comorbidity-defined groups. Outcomes included misclassification, diagnostic agreement, prevalence, sex distribution, disease-activity spillover, diagnostic delay, and clinical usability. Study designs included criteria papers, validation and concordance studies, population studies, health-system analyses, systematic reviews with criteria-relevant findings, and digital phenotyping studies.

Diagnostic accuracy and validation studies were appraised with QUADAS-2 domains, and observational studies were interpreted using ROBINS-I-informed domains, with attention to patient selection, index-test application, reference standard, flow and timing, confounding, missing data, and selective reporting.^15^^,^^16^ Because included records differed substantially in design, setting, thresholds, and reference standards, quantitative pooling was not attempted. Findings were synthesized narratively around recurrent error mechanisms rather than around a single pooled estimate.

## Results

**Figure 1.**
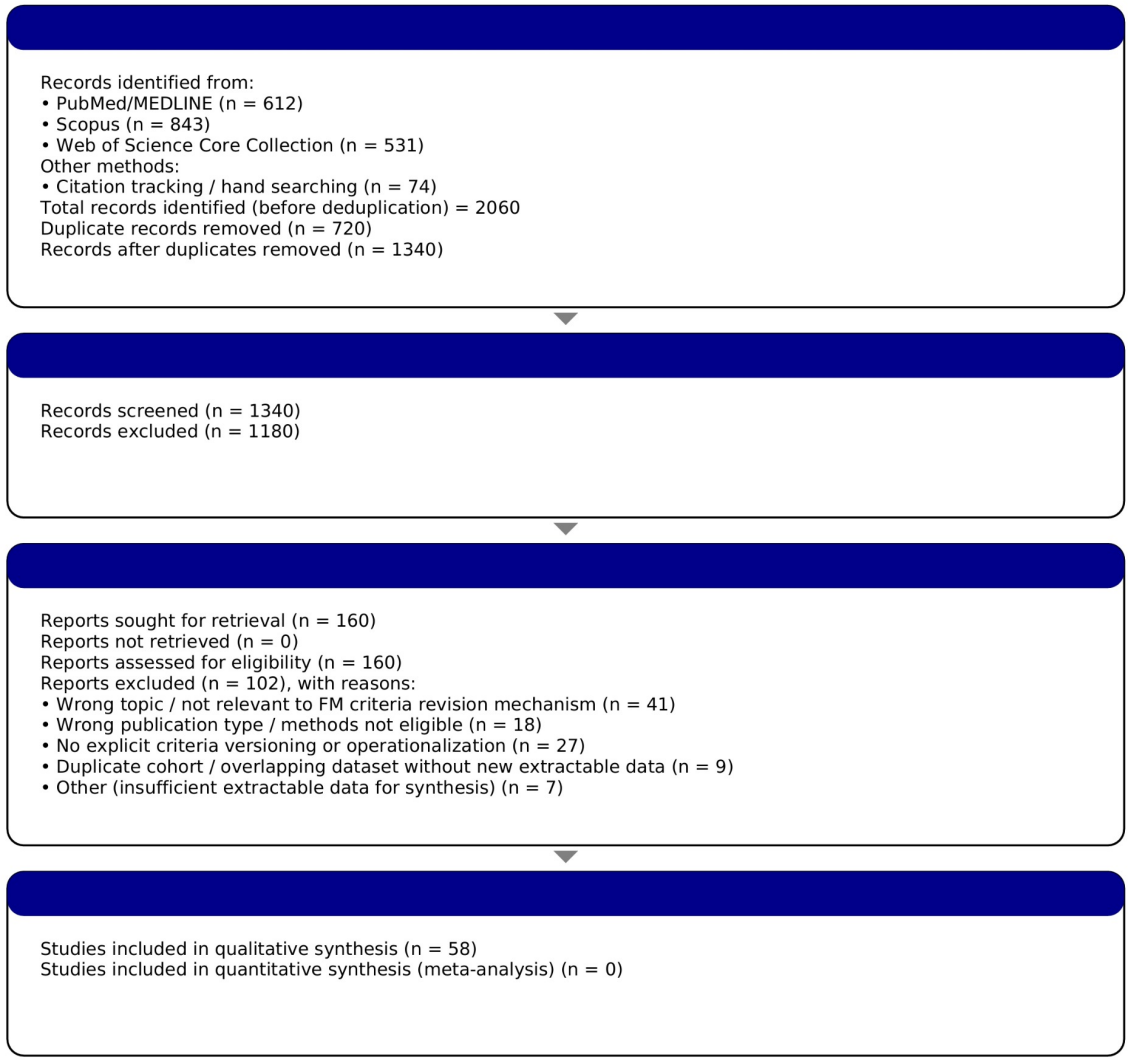
PRISMA 2020 flow diagram of study selection. The diagram summarizes database records, deduplication, title/abstract screening, full-text eligibility assessment, exclusion reasons, and final inclusion in qualitative synthesis (n = 58).

The search identified 2060 records before deduplication. After removal of 720 duplicates, 1340 records underwent title and abstract screening. One hundred sixty full-text reports were assessed, 102 were excluded with documented reasons, and 58 records were included in the qualitative synthesis. No quantitative meta-analysis was performed. The distribution of the 58 included records across the review questions is summarized in Table 1, and the PRISMA flow is shown in a Figure 1 (Supplementary Material).

**Figure 2.**
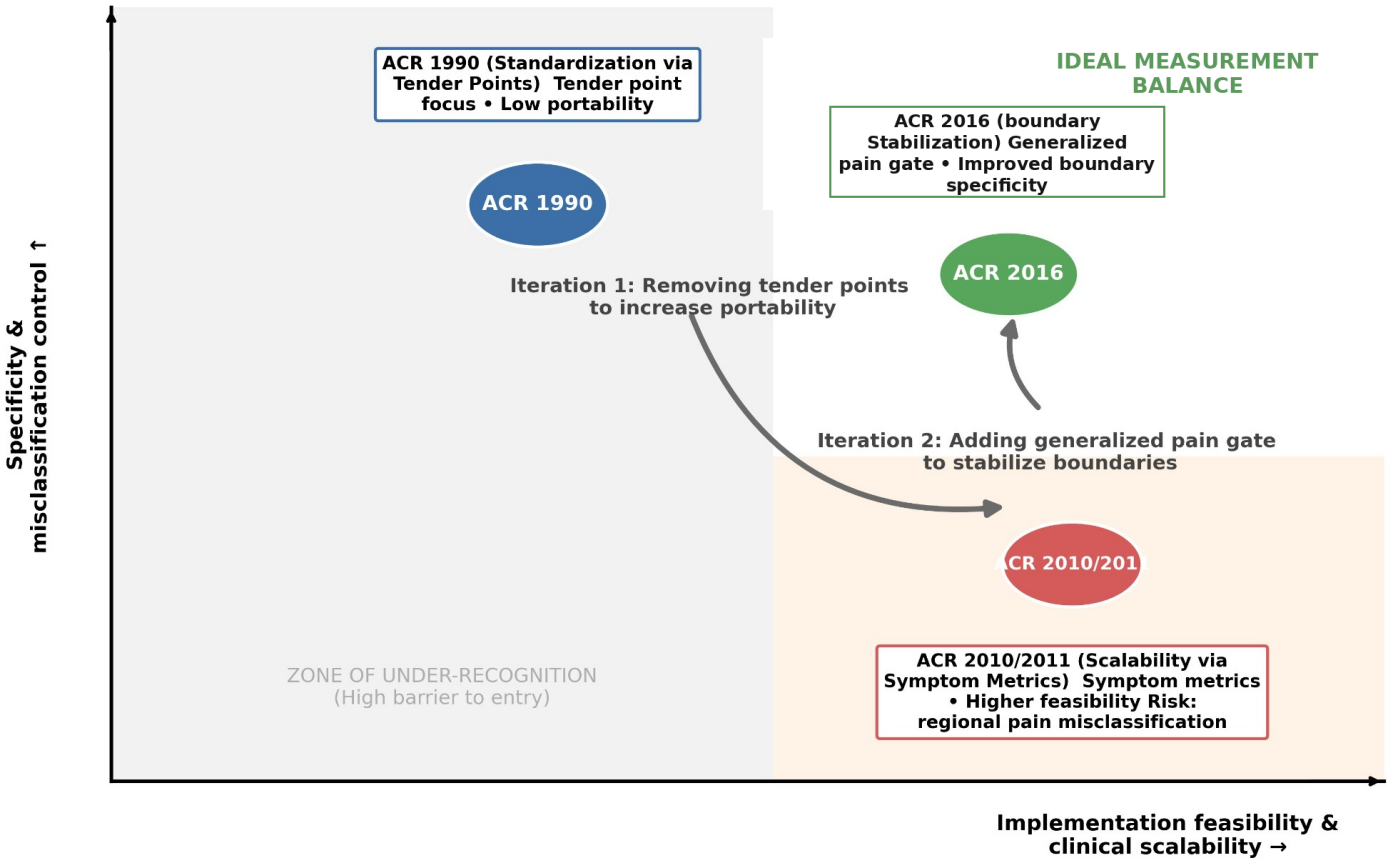
Fibromyalgia criteria evolution as multi-objective measurement engineering. The figure illustrates how criteria eras redistributed the balance between specificity, feasibility, portability, and misclassification control.

The evidence did not support a simple narrative in which newer criteria are always better. Instead, each criteria generation rebalanced feasibility, specificity, portability, and symptom coverage. The 1990 criteria improved research comparability but made trained tender-point examination central. The 2010 and 2011 criteria improved practicality and symptom coverage, but their portability also made them vulnerable to purpose mismatch when used as stand-alone clinical diagnosis. The 2016 criteria repaired part of this problem by requiring generalized pain and by clarifying that fibromyalgia can be diagnosed in the presence of other disorders, but residual boundary cases remain. This multi-objective evoluation of criteria is shown in Figure 2, and the corresponding criteria-era comparison is summarized in Table 2.

## Overdiagnosis, Misdiagnosis, and Missed Diagnosis Are Different Problems

Overdiagnosis is most likely when fibromyalgia is used as a label for persistent pain without verifying generalized pain distribution and symptom pattern. This can occur in regional musculoskeletal pain, osteoarthritis, enthesitis-dominant spondyloarthritis, untreated sleep disorder, endocrine disease, or high psychosocial distress. Body-map assessment and explicit chronic widespread pain rules are therefore not cosmetic additions; they are safeguards against counting regional or multifocal pain as fibromyalgia.^17^^,^^18^

Misdiagnosis is different. It occurs when the fibromyalgia label prematurely closes the diagnostic process and causes a treatable alternative to be missed. This is why the correct lesson from modern criteria is not “no tests are needed,” but rather “proportionate testing should follow clinical signals.” Objective synovitis, inflammatory markers, neurologic deficit, anemia, thyroid disease, infection, malignancy, myopathy, inflammatory back pain, or marked sleep-apnea risk should not be ignored simply because WPI/SSS thresholds are met.

Missed diagnosis is equally important. In a university-clinic study, criteria-based and clinician-based diagnoses disagreed substantially, showing that both false positives and false negatives occur in real practice.^19^ The under-, over-, and misdiagnosis literature similarly emphasizes that delayed or missed recognition may coexist with over-labeling in different settings.^20^ Population data add a further warning: many criteria-positive individuals report other diagnoses, while many people reporting a clinician diagnosis do not satisfy survey criteria.^21^^,^^22^ These findings do not prove that either clinicians or criteria are simply wrong; they show that the reference standard is unstable and context dependent. The diagnostic error typology is summarized in Table 3, and the main misclassification channels and rheumatology safeguards are illustrated in Figure 4.

Sex is a major contributor to missed diagnosis. The traditional view that fibromyalgia is overwhelmingly female was amplified by tender-point-based and referral-based ascertainment. When less selection-biased population approaches are used, the female predominance narrows substantially, suggesting that male fibromyalgia has often been under-recognized rather than biologically absent.^23^

## Do the 2016 Criteria Meet Clinical Need?

The 2016 criteria are a meaningful improvement because they added a generalized pain requirement, reduced misclassification of regional pain, and removed the confusing implication that fibromyalgia should be diagnosed only after excluding every other disorder. In this sense, they meet an important clinical need: they provide a common language that is feasible, reproducible, and more comorbidity-aware than earlier criteria.^4^

They are not, however, fully satisfactory as a complete clinical diagnostic system. First, they remain threshold instruments. Patients near the WPI/SSS boundary may move across the diagnostic line with small changes in symptom reporting, analgesic use, sleep status, or comorbidity control. Second, the self-report version is useful for research and screening but is not a substitute for clinician evaluation in a complex patient. Third, the criteria do not fully instruct clinicians how to handle borderline chronic widespread pain, male presentations, inflammatory comorbidity, vitamin D deficiency, or longitudinal reassessment.

Dimensional approaches help address this limitation. Polysymptomatic distress and fibromyalgia severity measures capture a continuum of symptom burden that is often more useful clinically than a binary label alone.^24^ Validation studies in Norway and China support the practical utility of the 2011/2016 framework, but they also show that language, culture, setting, and threshold calibration can influence performance.^25^^,^^26^ Recent UK Biobank work further suggests that alternative symptom thresholds and pain-distribution definitions may improve case-finding performance in large populations, reinforcing that the 2016 criteria are strong but not the final word.^27^

ACTION-American Pain Taxonomy (AAPT) diagnostic criteria, the Royal College of Physicians guideline, and European Alliance of Associations for Rheumatology (EULAR) recommendations each push the field in a similar direction: fibromyalgia should be recognized positively, interpreted multidimensionally, and managed through education, exercise, sleep optimization, psychological strategies, and symptom-targeted pharmacotherapy when appropriate.^28^^-^^30^ Health-system analyses showing changing diagnostic patterns after criteria revisions also caution that criteria do not merely detect prevalence; they shape clinical labeling.^^31^ The adequacy of the 2016 criteria in current clinical practice is summarized in Table 4.^

## Chronic Widespread Pain Should Not Be Equated with Fibromyalgia

Chronic widespread pain is primarily a distributional construct. Fibromyalgia adds a characteristic symptom profile and severity burden. The distinction matters because a patient may have chronic widespread pain due to inflammatory disease, osteoarthritis, endocrine disorder, hypermobility, neuropathic pain, myopathy, sleep disorder, or mixed pain mechanisms without satisfying a fibromyalgia phenotype. Conversely, a patient may meet fibromyalgia criteria while also having another active disorder.

**Figure 3.**
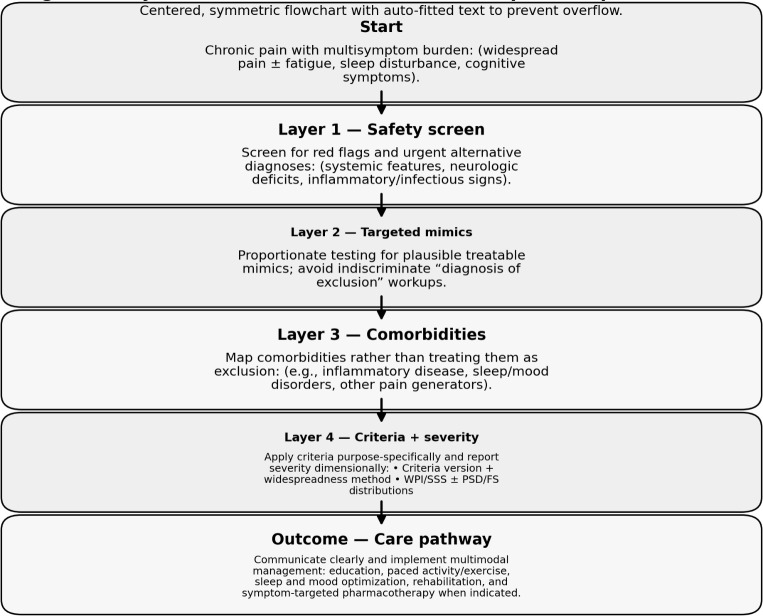
Layered assessment workflow for fibromyalgia-spectrum presentations. The workflow emphasizes safety screening, targeted exclusion of plausible mimics, comorbidity mapping, purpose-specific criteria application, dimensional severity reporting, and care-pathway planning.

**Figure 4.**
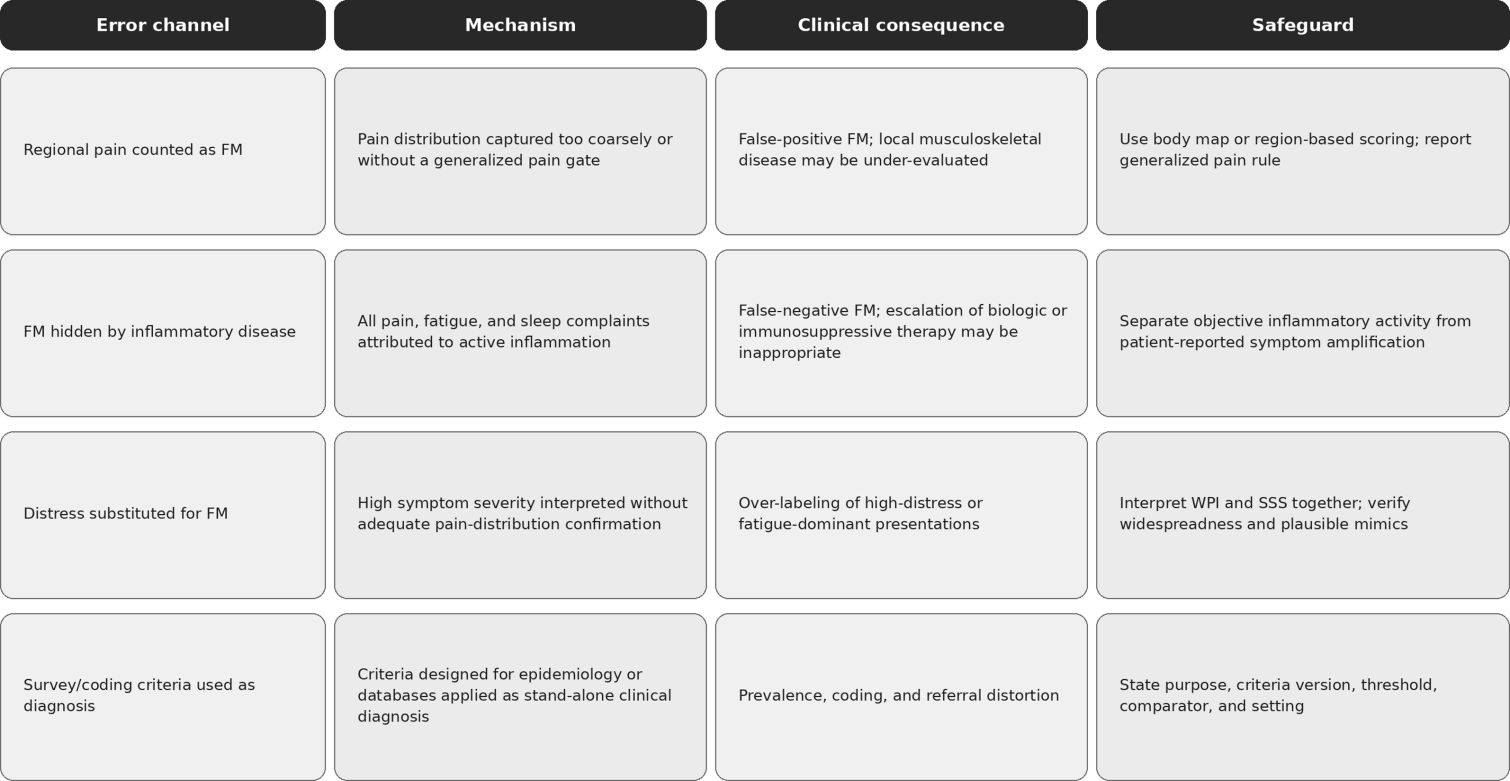
Misclassification channels and rheumatology safeguards. The figure summarizes false-positive and false-negative pathways that arise when pain distribution, symptom severity, comorbidity, and criteria purpose are not reported together.

A practical distinction requires 4 steps: confirm pain chronicity; map distribution using a body map or equivalent structured method; apply WPI/SSS and generalized pain requirements; and interpret symptoms in relation to comorbid disease activity, objective inflammation, sleep quality, mood symptoms, medications, and treatable metabolic abnormalities. The result should be 1 of 5 clinically useful outputs: fibromyalgia alone, fibromyalgia with comorbidity, chronic widespread pain without fibromyalgia, alternative diagnosis, or uncertain presentation requiring reassessment. This layered assessment workflow is shown in Figure 3, and the practical separation of fibromyalgia from chronic widespread pain is presented in Table 5.

## Prevalence Variability and the Narrowed Female-to-Male Ratio

The wide prevalence range reported in fibromyalgia is not simply epidemiologic noise. It reflects criteria generation, sampling frame, method of pain distribution capture, case-finding strategy, cultural and language factors, and whether studies count clinician-diagnosed cases or criteria-positive cases. Studies comparing ACR 1990, ACR 2010, and modified 2010 definitions show that prevalence changes when tender points are removed and symptom-based thresholds are used.^32^ European surveys, population-based medical record studies, worldwide epidemiologic reviews, and meta-analyses all show that prevalence estimates occupy a broad range because the measurement architecture is not constant across studies.^33^^-^^38^

The narrowing of the female-to-male ratio after the 2010 criteria is best interpreted as an ascertainment effect rather than an abrupt biological change. Tender-point examination, referral patterns, and clinician expectations tended to magnify female predominance. Symptom-based criteria widened the doorway for men whose pain amplification, fatigue, sleep disturbance, and cognitive symptoms were present but not captured by tender-point thresholds or were interpreted through different clinical stereotypes. This does not mean that current criteria work perfectly in men. Men may still be missed if clinicians expect a female phenotype, if men under-report fatigue or affective distress, or if occupational/regional pain is not mapped carefully. The solution is not a separate male criterion set but sex-sensitive screening, explicit body mapping, and dimensional severity reporting.

## Comorbid Rheumatic Disease and Diagnostic Delay

Diagnostic delay is common because fibromyalgia symptoms are often reattributed to other disorders, while other disorders may be reattributed to fibromyalgia. Patient surveys show that the journey to diagnosis can be long and fragmented, with substantial healthcare use before explanation and management become coherent.^39^ In rheumatology, the challenge is greatest when fibromyalgia coexists with inflammatory arthritis or spondyloarthritis.

In spondyloarthritis, concomitant fibromyalgia can inflate patient-reported disease activity, fatigue, pain, and function scores, complicating the interpretation of Bath Ankylosing Spondylitis Disease Activity Index (BASDAI), Ankylosing Spondylitis Disease Activity Score (ASDAS)-adjacent constructs, enthesitis, and global assessments.^40^^-^^42^ In inflammatory arthritis more broadly, systematic reviews show that comorbid fibromyalgia is common and clinically important.^43^^,^^44^ In rheumatoid arthritis, fibromyalgia may be frequent but unrelated to inflammatory activity, and it can complicate disease management by raising tender-joint counts and patient global scores.^45^^-^^47^ Similar effects are reported in psoriatic arthritis, where coexisting fibromyalgia can worsen clinical disease-activity indices without necessarily indicating equivalent objective inflammation.^48^^,^^49^

**Figure 5.**
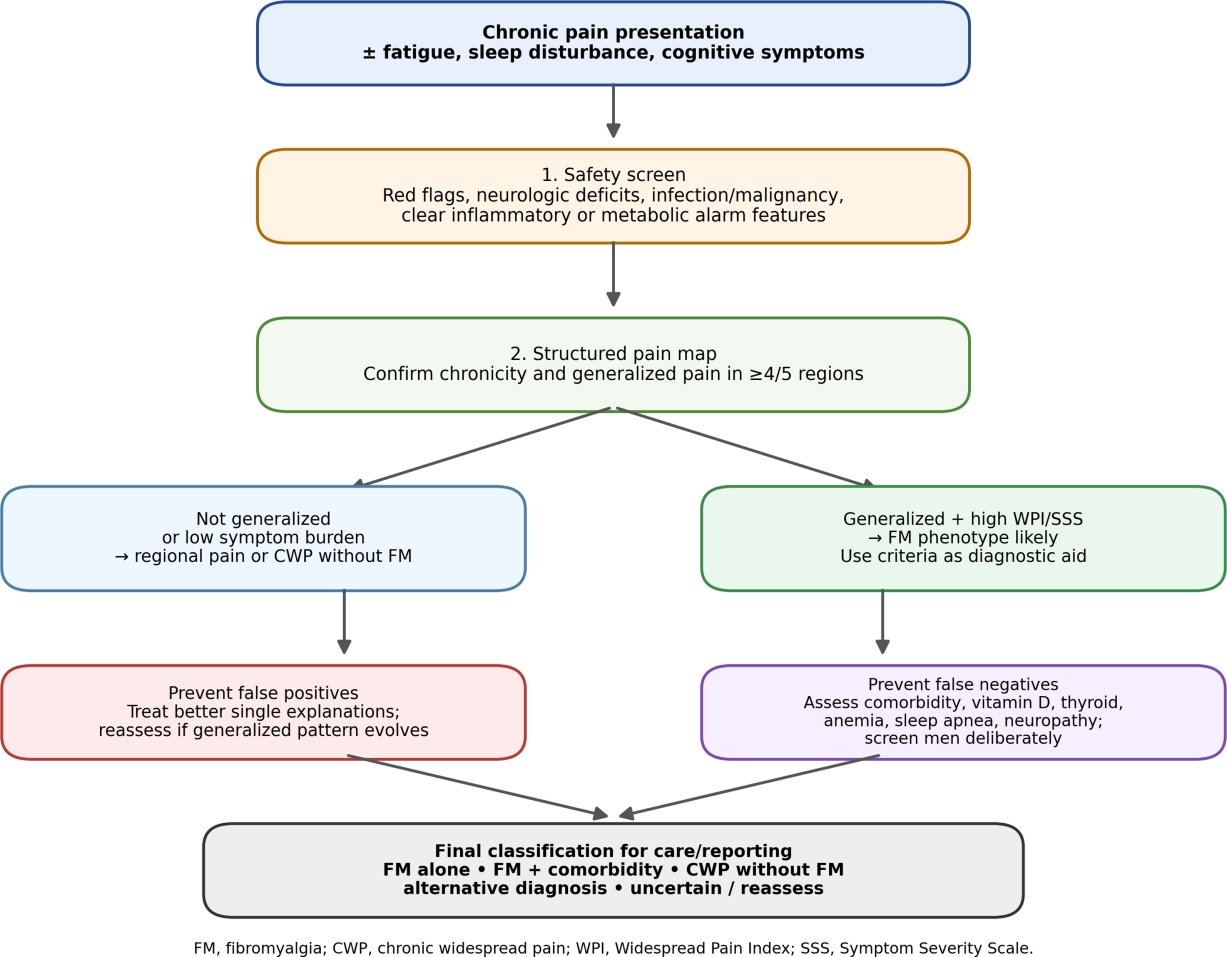
Diagnostic decision matrix for reducing overdiagnosis, misdiagnosis, and missed fibromyalgia. The diagram shows how chronic pain presentations can be triaged through safety screening, body-map verification, WPI/SSS scoring, comorbidity assessment, vitamin D and other mimic/modifier evaluation, sex-sensitive screening, and final classification.

The practical response is a layered model. First, determine whether objective inflammatory activity is present using swollen-joint count, CRP/ESR, imaging, or other disease-specific indicators. Second, quantify fibromyalgia-spectrum symptom burden separately with WPI/SSS, Polysymptomatic Distress (PSD), or Revised Fibromyalgia Impact Questionnaire (FIQR). Third, avoid escalating immunosuppression solely because pain, fatigue, patient global or tender-joint count is high when objective inflammation is controlled. Fourth, reassess after treating active inflammation, sleep disorder, mood disorder, vitamin D deficiency, or other modifiable contributors. Centralized pain in rheumatoid arthritis and related conditions should be viewed as a parallel treatment target, not as proof that inflammatory disease is inactive or active by itself.^50 A diagnostic decision matrix for reducing overdiagnosis, misdiagnosis, and missed fibromyalgia is presented in Figure 5.^

### Digital Tools, Vitamin D Deficiency, and Other Modifiable Contributors

Digital pain drawings, clustering methods, and decision-support tools may reduce missed diagnosis by making pain distribution and symptom patterns more visible. Pain-drawing clustering has identified undiagnosed fibromyalgia-pattern presentations, while large-language-model and neural/clinical approaches illustrate how computational tools may assist recognition.^51^^-^^53^ Their role should be supportive rather than determinative. Algorithms trained on noisy labels may reproduce overdiagnosis or sex bias unless body-map quality, comorbidity status, and symptom severity are explicitly modeled.

General fibromyalgia management literature and instruments such as the FIQR support the same principle: diagnosis should not end with a binary label; it should lead to structured severity measurement and care planning.^54^^,^^55^ This is particularly relevant when vitamin D deficiency is present. Vitamin D deficiency is common in many communities and may contribute to musculoskeletal pain, fatigue, and low physical capacity. However, it should not automatically replace fibromyalgia as the diagnosis when the fibromyalgia phenotype is otherwise present.

The most defensible approach is to treat vitamin D deficiency as a potentially modifiable mimic or symptom modifier. If pain is focal or metabolic-bone features dominate, vitamin D deficiency may be the primary explanation. If generalized pain, WPI/SSS thresholds, sleep disturbance, fatigue, and cognitive symptoms persist after appropriate replacement or are disproportionate to the deficiency, fibromyalgia can be diagnosed concurrently. Systematic reviews on vitamin D in fibromyalgia support checking and correcting deficiency while also warning that the evidence is not strong enough to treat vitamin D status as a stand-alone diagnostic discriminator.^56^^-^^^58^[Table t6-ar-41-3-155] Common diagnostic dilemmas and recommended safeguards are summarized in Table 6.^

## Discussion

The main finding of this review is that fibromyalgia criteria keep changing because the construct is measured under persistent uncertainty. Revisions are not signs of repeated failure; they are attempts to recalibrate the balance between research standardization, clinical feasibility, symptom coverage, and misclassification control. The 1990 criteria gave fibromyalgia a reproducible research identity, the 2010/2011 criteria made the syndrome more portable and symptom-centered, and the 2016 revision improved boundary discipline through a generalized pain gate and comorbidity compatibility.

Yet the 2016 criteria should not be considered fully sufficient. They are adequate as a common framework and a useful clinical aid, but not as a complete diagnostic pathway. They do not by themselves decide how much testing is appropriate, how to separate chronic widespread pain from fibromyalgia in borderline cases, how to handle vitamin D deficiency, how to prevent disease-activity spillover in inflammatory arthritis, or how to reduce male under-recognition. These tasks require a clinical algorithm layered onto the criteria.

**Figure 6.**
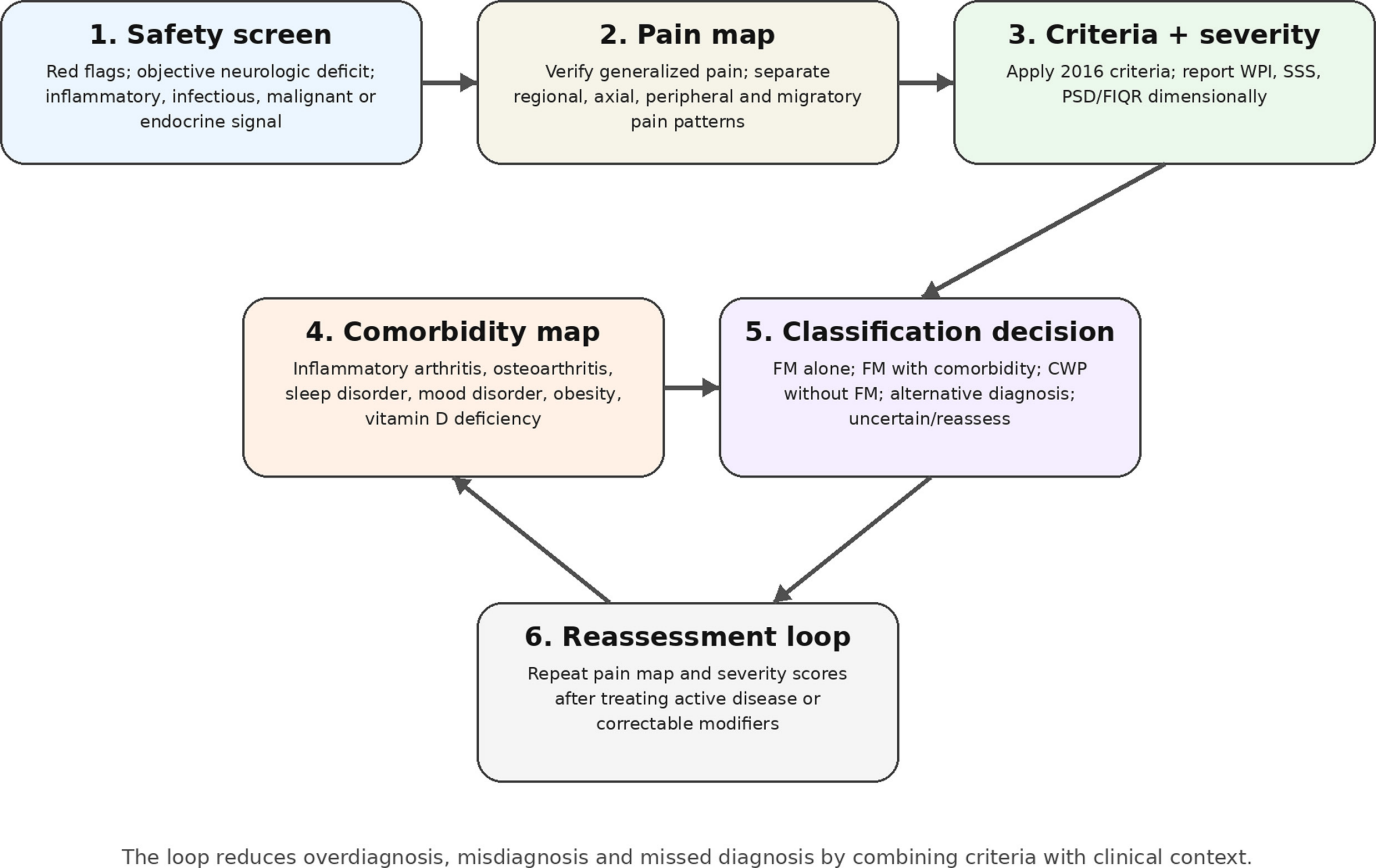
Diagnostic safeguard loop for fibromyalgia-spectrum assessment. The loop integrates safety screening, body-map verification, WPI/SSS and dimensional severity, comorbidity mapping, classification decision, and reassessment after treatment of active disease or correctable modifiers.

New criteria may eventually be needed, but the field should avoid another simple threshold-only revision. The next generation should be purpose-specific: 1 form for research classification, 1 for population case-finding, and 1 for clinical decision support. It should require structured pain-distribution capture, retain dimensional severity reporting, define how comorbidity and treatable modifiers are reported, and provide reassessment rules for borderline or mixed presentations. Such a system would not eliminate uncertainty, but it would make uncertainty transparent. The resulting diagnostic safeguard loop for fibromyalgia-spectrum assessment is illustrated in Figure 6.

For rheumatologists, the central practical message is to diagnose fibromyalgia positively but not casually. Positive diagnosis means recognizing a reproducible phenotype of generalized pain, symptom severity, and functional impact. Not casual means avoiding the use of fibromyalgia as a residual label for unexplained pain or as an explanation that blocks evaluation of objective inflammatory, neurologic, endocrine, infectious, or metabolic signals. A patient can have both fibromyalgia and another disorder, and the clinical task is to decide which component is active, treatable, measurable, and responsible for current disability.

Prevalence studies should therefore report the criteria version, pain-map method, threshold modifications, sex-specific estimates, sampling frame, clinician-diagnosed versus criteria-positive status, and comorbidity exclusions or inclusions. Without this minimum reporting set, prevalence differences may be misread as true biological differences when they are partly products of measurement architecture.

### Limitations

This review is limited by the heterogeneity of the included literature. The 58 included records contained criteria papers, validation studies, population studies, comorbidity analyses, health-system data, systematic reviews, and emerging digital-method papers. These bodies of evidence do not share a single reference standard, which is precisely why a pooled diagnostic accuracy estimate was not appropriate. Some studies relied on clinician diagnosis, some on survey criteria, and some on self-reported fibromyalgia. The synthesis should therefore be interpreted as a structured account of diagnostic mechanisms rather than a meta-analysis of pooled sensitivity or specificity.

A second limitation is that newer digital and AI-assisted studies are promising but remain at an early stage. Their inclusion helps identify future directions but should not be read as evidence that automated diagnosis is ready to replace clinical judgment. Finally, vitamin D literature is clinically relevant because deficiency is common and modifiable, but current evidence supports correction and reassessment rather than use of vitamin D status as a decisive diagnostic rule.

### Clinical implications

- Use the 2016 criteria as a structured diagnostic aid, not as a stand-alone substitute for clinical reasoning.
- Separate chronic widespread pain from fibromyalgia by combining body-map verification with WPI/SSS severity and comorbidity interpretation.
- Prevent overdiagnosis through generalized pain verification and targeted mimic evaluation.
- Prevent missed diagnosis in men and comorbid rheumatic disease by applying criteria consistently and reporting dimensional severity.
- Treat vitamin D deficiency and other modifiable contributors, but do not let them automatically exclude fibromyalgia when the full phenotype persists.

## Conclusion

Fibromyalgia criteria have evolved because the syndrome lies at the intersection of chronic widespread pain, nociplastic mechanisms, multisymptom burden, and imperfect measurement. The 2016 criteria provide the strongest current common language for practice and research, but they are not fully sufficient for all clinical needs. Their greatest strength is improved boundary control; their main limitation is that they do not fully address comorbidity, sex-sensitive under-recognition, vitamin D attribution errors, longitudinal reassessment, or the distinction between classification and diagnosis.

Future criteria should not simply raise or lower thresholds. They should integrate generalized pain verification, dimensional severity, comorbidity-aware interpretation, sex-sensitive screening, and explicit separation of chronic widespread pain from fibromyalgia. Until then, the most reliable clinical approach is a layered workflow: safety screen, pain map, criteria application, comorbidity and modifier assessment, dimensional severity reporting, and reassessment when the presentation is borderline or mixed.

## Data Availability Statement:

The data that support the findings of this study are available on request from the corresponding author.

## Artificial Intelligence Usage Statement:

The author declares that this study used ChatGPT (Open AI) to improve language and grammar during the preparation of the manuscript.

## Peer-review:

Externally peer-reviewed.

## Author Contributions:

Concept – A.G.; Design – A.G.; Supervision – A.G.; Resources – A.G.; Materials – A.G.; Data Collection and/or Processing – A.G.; Analysis and/or Interpretation – A.G.; Literature Search – A.G.; Writing – A.G.; Critical Review – A.G.

## Declaration of Interests:

The author has no conflicts of interest to declare.

## Funding:

The author declared that this study has received no financial support.

## Supplementary Materials

Supplementary Material

## Figures and Tables

**Table 1.** Evidence Map Linking the PRISMA 58 Included Records to the Main Diagnostic Questions

Evidence Domain	Main Contribution	References
Criteria architecture and diagnostic construct	ACR 1990/2010/2011/2016, AAPT, ICD-11/nociplastic framing, criteria-vs.-diagnosis distinction	^1^^-^13,^28^^-^^30^
Review methods and appraisal	PRISMA reporting, QUADAS-2, ROBINS-I-informed appraisal	^14^,^-^^16^
Pain distribution, CWP boundary, and body mapping	Generalized pain rule, revised CWP criteria, pain drawings/body maps	^17^,^18^,^51^
Overdiagnosis, misdiagnosis, missed diagnosis	Clinician-criteria disagreement, NHIS mislabeling, under/over/misdiagnosis review	^19^-^23^
Validation and criteria optimization	Dimensional PSD, Norway/China validation, UK Biobank criteria optimization	^24^ ^-^ ^27^
Prevalence and sex ratio	ACR-era comparisons, population surveys, prevalence updates, and meta-analyses	^31^ ^-^ ^38^
Comorbidity and rheumatology spillover	SpA, RA, PsA, disease-activity inflation, biologic-use implications	^39^^-^ ^50^
Digital tools and measurement support	Pain-drawing clustering, LLM/sentiment analysis, neural/clinical classifiers	^51^-^53^
Modifiable mimics and symptom modifiers	General FM management, FIQR, vitamin D testing/replacement and supplementation evidence	^54^ ^-^ ^58^

AAPT, ACTION-American Pain Taxonomy; ACR, American College of Rheumatology; ASDAS, Ankylosing Spondylitis Disease Activity Score; BASDAI, Bath Ankylosing Spondylitis Disease Activity Index; EULAR, European Alliance of Association for Rheumatology; FIQR, Revised Fibromyalgia Impact Questionnaire; FM, fibromyalgia; IASP, International Association for the Study of Pain; ICD-11, International Classification of Diseases, 11th Revision; PSD, polysymptomatic distress; SSS, Symptom Severity Index; WPI, Widespread Pain Index.

**Table 2.** Criteria Eras and the Diagnostic Problem Each Revision Attempted to Solve

Criteria Set	Core Structure	Main Gain	Residual Diagnostic Risk
ACR 1990	Chronic widespread pain plus ≥11/18 tender points	Research standardization and cohort homogeneity	Examiner dependence; limited fatigue/sleep/cognition capture; male and non-tender-point phenotypes may be missed
ACR 2010	WPI + SSS; symptom domains incorporated	Feasible symptom-based assessment	Coarse widespreadness; risk of over-including high-distress or regional pain cases
ACR 2011 survey modification	Self-reportable WPI/SSS operationalization	Epidemiologic portability	Useful for case finding, less secure for individual clinical diagnosis without examination
ACR 2016	Generalized pain in ≥4/5 regions plus WPI/SSS thresholds; comorbidity compatibility	Better boundary control and clearer coexistence logic	Still threshold-based; does not fully solve sex-sensitive under-recognition or comorbidity-related attribution errors
AAPT 2019	Multidimensional taxonomy with diagnostic criteria, common features, comorbidities, and consequences	More clinically descriptive	Less widely embedded in epidemiologic surveillance and routine coding

ACR, American College of Rheumatology.

**Table 3.** Diagnostic Error Typology in Fibromyalgia-spectrum Presentations

Diagnostic Harm	Typical Mechanism	Clinical Consequence	Practical Safeguard
Overdiagnosis	Regional pain, high distress, nonspecific fatigue, or untreated mimic counted as FM	False-positive FM label; unnecessary pain-focused pathway	Body map, generalized pain gate, red flags, targeted mimic evaluation
Misdiagnosis	FM label used before inflammatory, endocrine, neurologic, sleep or metabolic alternatives are assessed	Treatable condition missed or undertreated	Signal-driven tests rather than unlimited rule-out testing
Missed diagnosis	Clinician attribution to sex stereotype, inflammatory disease, obesity, vitamin D deficiency, mood disorder, or aging	Delayed explanation, repeated referrals, ongoing uncertainty	Sex-sensitive screening, WPI/SSS scoring, reassessment after comorbidity treatment
Diagnostic spillover	Comorbid FM inflates tender-joint count, pain Visual Analoge Scale, global scores, or fatigue	Overestimation of inflammatory activity and potential overtreatment	Separate objective inflammation from centralized pain burden

**Table 4.** Adequacy of the 2016 Criteria in Current Clinical Practice

Judgment	Reason
Yes	Generalized pain gate improves specificity and reduces regional-pain misclassification.
Yes	Explicit coexistence with other illnesses reduces the harmful “diagnosis of exclusion” model.
Partly	WPI/SSS thresholds are feasible, but boundary patients require dimensional interpretation.
Partly	Self-report tools help screening and research but cannot replace examination and comorbidity assessment.
No	The criteria do not fully solve male under-recognition, inflammatory-disease spillover, vitamin D attribution errors, or longitudinal reassessment rules.
Future need	New criteria should be purpose-specific, sex-sensitive, comorbidity-aware and linked to a body-map/severity reporting standard.

**Table 5.** Practical Separation of Fibromyalgia from Chronic Widespread Pain

Domain	Fibromyalgia	Chronic Widespread Pain Without Confirmed Fibromyalgia
Pain distribution	Generalized pain in required regions	Widespread pain may be present, but may not meet FM generalized pain gate or may reflect another disorder
Symptom severity	WPI/SSS threshold plus fatigue, sleep and cognitive symptom burden	Pain distribution dominates; systemic symptom burden may be absent or explained by another condition
Comorbidity logic	Can coexist with inflammatory, endocrine, metabolic or sleep disorders	May be secondary to active disease or mixed mechanisms
Clinical output	Positive FM phenotype after proportionate exclusion of red flags and treatable mimics	Descriptor requiring etiologic work-up and follow-up
Management implication	Education, exercise, sleep regulation, pacing, psychological and symptom-targeted treatment	Treat cause or contributors first; reassess phenotype over time

**Table 6. t6-ar-41-3-155:** Handling Common Diagnostic Dilemmas in Fibromyalgia-spectrum Assessment

Clinical Situation	Recommended Diagnostic Stance	Primary Safeguard
Vitamin D deficiency with focal bone/muscle pain and low FM symptom burden	Treat deficiency; reassess before applying FM label	Avoid overdiagnosis
Vitamin D deficiency plus generalized pain and high WPI/SSS phenotype	Correct deficiency but diagnose FM if criteria and clinical pattern are met	Avoid missed diagnosis
Inflammatory arthritis with high tender-joint count and high patient global but low objective inflammation	Assess FM/centralized pain separately before escalating immunosuppression	Avoid overtreatment
Male patient with fatigue, sleep disturbance, and widespread pain but no tender-point emphasis	Use 2016 criteria and body map rather than sex stereotype	Avoid male under-recognition
Borderline WPI/SSS or unstable distribution	Report dimensional severity and reassess longitudinally	Avoid premature closure
