# Supplementary material for: Why Fibromyalgia Criteria Keep Changing: A Systematic Review of Misclassification, Measurement, and Diagnostic Spillover
Source: Arch Rheumatol. 2026 Jul 6;41(3):155–67. doi: 10.5152/ArchRheumatol.2026.26498 (PMC13401112; doi:10.5152/ArchRheumatol.2026.26498)
Supplement: Supplementary Material [file supplementary_material.pdf]

**Supplementary Table 1.** PRISMA accounting	
**Step**	**Number**
Records identified before deduplication	2060
Duplicates removed	720
Records screened	1340
Records excluded at title/abstract level	1180
Full texts assessed	160
Full texts excluded	102
Studies included in qualitative synthesis	58
Studies included in quantitative synthesis/meta-analysis	0

Supplementary File 1

Review title

Why Fibromyalgia Criteria Keep Changing: A Systematic Review of Misclassification, Measurement, and Diagnostic Spillover in Rheumatology Practice

Databases and dates

Databases searched: MEDLINE/PubMed, Scopus, and Web of Science Core Collection. Time window: January 1, 1990 to March 4, 2026. Last search date: March 4, 2026. Additional methods included backward and forward citation tracking of core criteria papers and major validation studies, plus targeted hand searching of health system and body map/digital phenotyping papers.

Full search strings

PubMed/MEDLINE

("fibromyalgia"[Title/Abstract] OR Fibromyalgia[MeSH Terms]) AND ("criteria"[Title/Abstract] OR "criterion"[Title/Abstract] OR "classification"[Title/Abstract] OR "diagnostic"[Title/Abstract] OR "ACR"[Title/Abstract] OR "American College of Rheumatology"[Title/Abstract] OR "WPI"[Title/Abstract] OR "SSS"[Title/Abstract] OR "symptom severity"[Title/Abstract] OR "polysymptomatic distress"[Title/Abstract] OR "generalized pain"[Title/Abstract] OR "chronic wide-spread pain"[Title/Abstract] OR "body map"[Title/Abstract] OR "pain drawing"[Title/Abstract]) AND ("validation"[Title/Abstract] OR "agreement"[Title/Abstract] OR "kappa"[Title/Abstract] OR "misclassification"[Title/Abstract] OR "false positive"[Title/Abstract] OR "false negative"[Title/Abstract] OR "prevalence"[Title/Abstract] OR "sex"[Title/Abstract] OR "gender"[Title/Abstract] OR "registry"[Title/Abstract] OR "machine learning"[Title/Abstract] OR "nociplastic"[Title/Abstract])

Scopus

TITLE-ABS-KEY (fibromyalgia AND (criter* OR classification OR diagnostic OR ACR OR "American College of Rheumatology" OR WPI OR SSS OR "symptom severity" OR "polysymptomatic distress" OR "generalized pain" OR "chronic widespread pain" OR "body map" OR "pain drawing") AND (validation OR agreement OR kappa OR misclassification OR "false positive" OR "false negative" OR prevalence OR sex OR gender OR registry OR "machine learning" OR nociplastic))

Web of Science Core Collection

**Supplementary Table 2.** Reasons for full-text exclusion	
**Reason**	**n**
Wrong topic/not relevant to fibromyalgia criteria revision mechanism	41
Wrong publication type or methods not eligible	18
No explicit criteria versioning or operationalization	27
Duplicate cohort/overlapping dataset without new extractable data	9
Other: insufficient extractable data for synthesis	7

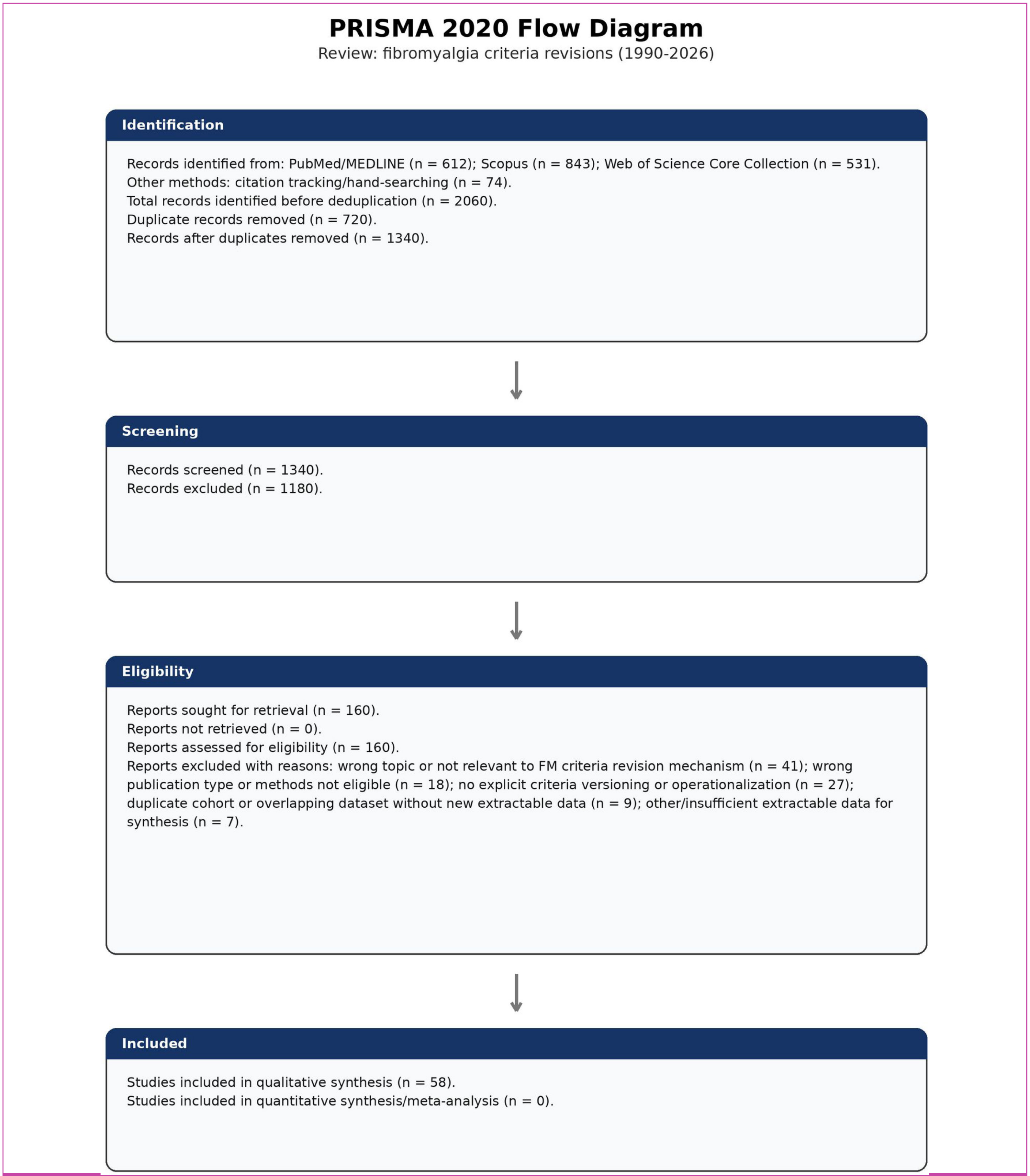

Supplementary Figure 1. PRISMA 2020 flow diagram used for the review.

TS=(fibromyalgia AND (criter* OR classification OR diagnostic OR ACR OR "American College of Rheumatology" OR WPI OR SSS OR "symptom severity" OR "polysymptomatic distress" OR "generalized pain" OR "chronic widespread pain" OR "body map" OR "pain drawing") AND (validation OR agreement OR kappa OR misclassification OR "false positive" OR "false negative" OR prevalence OR sex OR gender OR registry OR "machine learning" OR nociplastic))

Eligibility criteria

Inclusion criteria were primary criteria manuscripts and revisions; validation and concordance studies; studies evaluating prevalence or sex-ratio shifts across criteria; articles on differential-diagnosis spillover and comorbidity framing; health-system analyses; and digital/AI measurement studies linked to fibromyalgia case definition. Exclusion criteria were case reports and small case series without explicit criteria operationalization; narrative overviews without transparent selection methods; treatment trials without criteria-relevant data; and duplicate or overlapping cohorts without new extractable information.

Risk-of-bias approach

Diagnostic accuracy and validation studies were assessed with QUADAS-2 domains: patient selection, index test, reference standard, and flow and timing. Observational and health-system studies were reviewed with ROBINS-I-informed domains emphasizing confounding, selection bias, exposure classification, missing data, outcome measurement, and selective reporting. Because the corpus mixed criteria papers, validation studies, population studies, and health-system analyses, appraisal was used to guide interpretation rather than to generate pooled quality scores.
